# Weight Mobility and Obesity in a Representative Sample of the US Adult Population

**DOI:** 10.1155/2018/4561213

**Published:** 2018-06-10

**Authors:** Deanna J. M. Isaman, Amy E. Rothberg

**Affiliations:** ^1^Department of Biostatistics, University of Michigan, Ann Arbor, MI, USA; ^2^Department of Internal Medicine, University of Michigan, Ann Arbor, MI, USA; ^3^Department of Human Nutrition, University of Michigan, Ann Arbor, MI, USA

## Abstract

**Background:**

Despite the attention given to the prevalence of obesity, surprisingly little is known about the incidence or reduction of obesity. We report the 1-year incidence and remission of obesity in a representative sample of the US population.

**Methods:**

Individuals from the Medical Expenditure Panel Survey (MEPS) panel 17 were classified into standard obesity categories at enrollment and one year later. Incidence rates were calculated by age.

**Results:**

Although the overall prevalence of obesity remained nearly constant, remission rates from obesity (stratified by age) ranged from 11 to 27% while incidence rates ranged from 6 to 16%. For almost all age levels, the proportion of individuals leaving an obese or overweight state was greater than or equal to the proportion who progressed to a more severe level of overweight or obesity. Overall, 36% of adults lost at least 2.5 kg/m^2^ in the one-year period; only 8% gained 2.5 kg/m^2^ or more. Individuals less than 25 years of age had higher rates of leaving overweight (23% versus <16%) and obesity (27% versus 24%) classifications than people of other ages.

**Conclusions:**

Prevalence rates of obesity are well documented in the United States, but incidence is understudied. Public health efforts that target young people with overweight or obesity may yield the greatest benefit.

## 1. Background

Obesity is a significant health problem in the United States that has increased in urgency over time [1, 2]. Not only is obesity strongly associated with major causes of disability and death, but its prevalence has increased over the past thirty years, plateauing at over 30% of the US population and 37% worldwide [3–5].

Despite the attention given to the prevalence of obesity, surprisingly little is known about the incidence of obesity [6]. Longitudinal trends from the 1950s through 2008 have captured the changing dynamic of obesity over time in cohorts or subgroups [7–9], but do not capture individual-level change from year to year. Moreover, they miss the occurrence of weight loss on an individual level. This paucity of knowledge limits treatment paradigms, because individuals may gain or lose weight, even when, prevalence has plateaued on a national level and because understanding the characteristics associated with incidence or remission of obesity rates may suggest foci for support and resources.

In this paper, we report the annual incidence and reduction of obesity in an adult population using the Medical Expenditure Panel Survey (MEPS) from 2012 to 2013.

## 2. Methods

### 2.1. Study Population

Data from panel 17 (years 2012-2013) of the MEPS were used for this analysis. The MEPS is a panel survey of US noninstitutionalized residents, weighted to represent the US population. Key variables are measured repeatedly over a two-year period; body mass index (BMI), that is, the ratio of weight in kg to height in meters squared, was obtained one year apart during rounds 3 and 5. Panel 17 enrolled 17,923 individuals aged from birth to 85 years old. We limited our analysis to the adults (age at least 17, *N* = 12,799) who had BMI measurements available at both years (*N* = 11,935). Children were excluded due to the changing definitions of obesity between adolescence and adulthood which can create discrepancies in classification [10].

Analyses were run with and without including pregnant women. We present results without including pregnant women because the results were changed by less than 1%.

### 2.2. Outcomes of Interest

Our primary outcome of interest was the incidence or remission of three categories of excess weight. We defined four weight categories based on CDC guidelines: normal weight (BMI < 25 kg/m^2^), overweight (25 ≤ BMI< 30 kg/m^2^), class 1 obesity (30 ≤ BMI< 35 kg/m^2^), and class 2 or 3 obesity (BMI ≥ 35 kg/m^2^). For Asians, we defined weight categories using WHO and International Diabetes Federation (IDF) guidelines [11, 12] of normal weight (BMI < 23 kg/m^2^), overweight (23 ≤ BMI< 25 kg/m^2^), class 1 obesity (25 ≤ BMI< 30 kg/m^2^), and class 2 or 3 obesity (BMI ≥ 30 kg/m^2^).

We also investigated change in BMI by subtracting a difference over the one-year period. In addition, we defined a clinically meaningful change in BMI as 2.5 kg/m^2^ (half of a category width for overweight or obesity).

### 2.3. Age Classification

We defined age categories using 25-year spans: <25 years, 25–49 years, 50–74 years, and >75.

### 2.4. Statistical Analysis

All analyses used SAS 9.4 (SAS Institute Inc., Cary, NC, USA). We used the SURVEYFREQ procedure to report incidence, remission, and prevalence, adjusting for the MEPS sampling design. Average BMI is reported using the survey means procedure for individuals who progress or remit. These procedures weight the results to provide estimates that are representative of the US population in 2012-2013. Cells with 20 or fewer observations are not reported, due to concerns about the stability of the estimates.

To investigate the proportion of individuals who had meaningful gain or loss in BMI, we report the proportion of individuals (weighted based on the sampling design) who gained or lost more than 2.5 kg/m^2^. Considering more extreme weight changes, we also considered 5 kg/m^2^ or more.

Given the high retention rate for BMI, we report results for the adults who had BMI measurements available at both years. Analyses imputing BMI at follow-up based on baseline BMI showed no meaningful differences, with differences falling within rounding error.

## 3. Results

### 3.1. Demographic Descriptors and Nonresponse

Baseline demographic characteristics are displayed in Table 1. The retention rate for BMI was 93% (11,920/12,799). Ages ranged between 17 and 85 with a median of 43 years. The median BMI was 26.6 kg/m^2^, with first and 3rd quartiles of 23.6 and 31.2 kg/m^2^, respectively. Between baseline and follow-up, the mean (SD) change in BMI was 0.11 (0.04) kg/m^2^. In addition, the median change was −0.02 kg/m^2^ with the first and third quartiles of −0.9 and 1.1 kg/m^2^, respectively, suggesting that at least half of the respondents lost some weight during the year.

### 3.2. Change in BMI

To explore an individual's change in BMI as a function of age and obesity status, Table 2 displays the mean change in BMI based on obesity classification at baseline and follow-up for individuals in each of the four age groups. The block-diagonal entries reflect individuals who retained their obesity category between the two years. Individuals who retained their baseline obesity category had an absolute mean change in BMI less than 0.5 kg/m^2^, with standard deviations greater than 1. Despite this proximity to zero, the change was occasionally significant at the 0.05 level, generally for cells with large sample sizes over 600. Also, considering the number of statistical tests, some significant results may be spurious. Replication of these results would be necessary prior to concluding that mean weight change was nonzero in any of the weight-maintenance categories.

Among individuals who did not retain their baseline obesity classification, the average change in BMI was greater than 2.5 kg/m^2^, for all cells except transition from class 1 obesity to class 2 or 3 obesity in the older adults (ages 50–75). The BMI changes ranged from 0.1 to 8.0 kg/m^2^, except for class 2 or 3, where BMI could reach extreme values.

Investigating meaningful changes in BMI, we observed that 36% of individuals who reduced obesity categories lost at least 2.5 kg/m^2^, and only 8% of those who progressed to overweight or class 1 obesity gained 2.5 kg/m^2^ or more. Considering more extreme weight changes, 31% lost at least 5 kg/m^2^, while 3% gained 5 kg/m^2^ or more, when weighted to represent the US population.

### 3.3. Rates of Incidence and Reduction of Overweight and Obesity

Table 3 shows the proportion of people moving between weight categories from baseline to one year later, for four age categories, weighted to represent the US population. The block-diagonal entries represent the proportion of people in each age category who remained in their baseline weight category. For example, 85% of the young healthy-weight people retained a normal weight, 14% progressed to overweight, and less than 1% of young, healthy-weight individuals attained obesity at any classification level. The stable-weight rates ranged from 53% in the young obese to 85% in the healthy young, and individuals rarely progressed more than a single category of obesity in a single year. Among overweight and obese individuals, the youngest adults displayed higher weight mobility than other ages. The oldest individuals also displayed weight mobility, often lowering obesity status at higher rates than middle-aged individuals.

Reading the table rows left or right from the diagonal shows the progression rates to normal weight or overweight/obese, respectively. The progression rates into a lower weight category were higher than the progression rate to a higher weight category for all baseline weight categories, with one exception (individuals 50–75 years old who were overweight at baseline). Despite this downward weight mobility, the overall prevalence remained stable due to the upward weight mobility in other weight categories. For example, 43% of the overweight population was 25–49 years old, of which 14% moved to class 1 obesity which represents a total of 6% (43% times 14%) of the entire overweight population progressing. In contrast, only 9% of the overweight population was young at baseline, of which 23% moved to a normal weight, so these younger individuals who lost weight represent only 2% (9% times 23%) of the entire overweight population. Thus, the low rate of incident obesity in a large subpopulation contributes more to the total health status than a higher rate of remission in a smaller subpopulation.

## 4. Discussion

Our results suggest a need to look beyond population-level prevalence to consider the role of individuals in public health. Investigation of incident obesity in a population-based study provides opportunities to target subpopulations who are most likely to benefit from public health intervention. Our results suggest that young adults with overweight or obesity are more weight-mobile than other ages. As such, public health interventions that target young adults may be particularly effective.

Children and young adults, in particular, are a high-risk subpopulation. Obesity in youth not only predicts further obesity [6, 10], but is also associated with earlier onset of type 2 diabetes mellitus [13], higher risk for complications and comorbidities [14], and higher mortality [15] compared to obesity that develops as an adult. Thus, targeted disruption or delay in the progression of obesity for this young-adult, weight-mobile subpopulation can produce a large benefit to individuals, employers, health-care payers, and society [9].

More importantly, our results suggest that the observed reduction in obesity category is not a trivial drop across an arbitrary threshold. Instead, we found that obesity reduction, when it occurred, was associated with meaningful decreases in BMI. The average BMI change among these weight-mobile individuals was typically greater than half the width of an obesity category. That is, some individuals had a small change, moving just over the threshold of an obesity category whereas others moved from the high end of one category to the low end of a lower weight. To put the mean weight change (gained or lost) in context, for someone who is 160 cm (5 ft. 3 in.) tall with an initial BMI of 35 kg/m^2^, a 2.5 kg/m^2^ drop in BMI equates to a 7% drop in body weight. Taller or lighter individuals would generate a larger percentage decrease in weight for the same change in BMI. If the 160 cm individual in this example started at an initial BMI of 50 kg/m^2^, the percent weight loss would be 5%. For comparison, the Diabetes Prevention Program (DPP) set a 7% weight-loss goal for participants to decrease their risk of diabetes, complications, and comorbidities [16]. Similarly, the CDC and NIH recommend a 5–10% weight-loss goal for reducing risk [17]. Thus, our results show that over a third of the individuals who reduced their obesity classification, lost recommended levels of weight. In contrast, among the individuals who gained weight, less than 10% gained substantial amounts of weight in this one-year period. So, although the averages among increasers and reducers were the same, the distributions of weight change were very different.

Another surprising result was that for almost every age category and baseline obesity category, the proportion of individuals reducing their classification status was greater than those who increased their classification. For example, among the young adults, 23% moved from overweight to normal weight, while only 14% of young adults progressed from normal weight to overweight. This is surprising because the overall prevalence did not change substantially. However, a careful inspection revealed that this apparent dichotomy was due to the prevalence of various subgroups. Continuing our example with young adults, there were more than twice as many individuals who started in healthy weight as opposed to overweight; so the total number of young adults moving between the two categories was comparable. By targeting the weight-mobile young adults, the incidence may be reduced which would lead to decreased prevalence.

The primary limitation of this study is the one year of follow-up in the MEPS. Epidemiologic data are needed to understand the characteristics of individuals who attain a substantial reduction in BMI, and to interpret the weight reduction in the oldest individuals, as it may be the result of a deterioration in health [18, 19]. However, any longitudinal study, by design, would not provide up-to-date reports on incidence. In contrast, a MEPS cohort can provide current incidence estimates. As such, an epidemiologic study and a MEPS cohort complement each other, in the same way that our incidence study supplements the national prevalence reports. The complementary views provide a nuanced understanding of obesity.

## 5. Conclusion

In conclusion, although the prevalence of obesity is stable, individual weight loss and gain is not static. More research should focus on understanding the drivers of incidence and remission of obesity, with a goal of facilitating weight loss to prevent disease or reduce the burden of comorbid health conditions. In particular, prevention efforts should target young adults less than 25 years old with overweight and obesity, as they are most likely to achieve reduction in obesity status.

## Figures and Tables

**Table 1:** Demographic characteristics at baseline.

		Complete data *N* = 11,920		Missing data *N* = 879	
BMI (kg/m^2^)	Mean (SE)	27.8	(0.10)	28.4	(0.28)

Age (y)	Mean (SE)	46.2	(0.28)	47.8	(1.2)

Sex	(% male)	46.4%		43.1%	

Diabetes	(%)	10.1%		11.5%	

Poverty index	(%)				
	Poor	15.4%		15.2%	
	Near poor	5.6%		9.1%	
	Low income	14.8%		17.5%	
	Middle income	29.7%		33.2%	
	High income	33.1%		25.0%	

Race	(%)				
	Hispanic	17.3%		21.4%	
	White	62.5%		59.2%	
	Black	12.1%		13.1%	
	Asian	5.3%		4.4%	
	Other or multiple	2.9%		1.9%	

**Table 2:** Mean (SD) change in BMI in one year by progression and age category.

Baseline (2012)	One-year follow-up (2013)			
	Normal weight	Overweight	Class 1	Class 2 or 3
Normal weight				
Age < 25	0.18 (1.8)	3.70 (2.0)	∗	∗
Age 25–49	0.01 (1.5)^∗∗^	2.67 (1.9)	7.6 (3.2)	∗
Age 50–74	−0.12 (1.4)	2.54 (1.9)	8.2 (3.5)	∗
Age > 75	−0.10 (1.5)^∗∗^	2.89 (1.8)	∗	∗
Overweight				
Age < 25	−3.35 (2.2)	0.09 (1.5)^∗∗^	3.32 (1.7)	∗
Age 25–49	−2.86 (2.1)	0.05 (1.3)^∗∗^	2.83 (1.6)	10.8 (5.3)
Age 50–74	−2.81 (2.3)	−0.02 (1.2)^∗∗^	2.66 (1.7)	9.9 (3.8)
Age > 75	−2.69 (1.4)	−0.20 (1.4)^∗∗^	2.73 (1.7)	∗
Class 1				
Age < 25	∗	−3.54 (2.0)	0.14 (1.7)^∗∗^	4.41 (2.6)
Age 25–49	∗	−2.96 (1.8)	0.12 (1.4)	4.54 (3.7)
Age 50–74	∗	−2.66 (1.6)	0.03 (1.4)^∗∗^	3.71 (2.4)
Age > 75	∗	−2.47 (1.6)	−0.16 (1.2)^∗∗^	∗
Class 2 or 3				
Age < 25	∗	∗	−4.16 (2.6)	0.43 (4.0)^∗^
Age 25–49	∗	−11.3 (6.2)	−4.3 (3.5)	0.44 (3.8)
Age 50–74	∗	∗	−3.86 (3.3)	0.26 (3.8)^∗^
Age > 75	∗	∗	−2.92 (2.1)	−0.28 (3.7)^∗^

^∗^Cell count 20 or less. ^∗∗^Not significantly different from zero (*P* > 0.05).

**Table 3:** Rates of incidence and reduction of overweight and obesity from baseline to follow-up.

Baseline (2012)	One-year follow-up (2013)				
	Normal weight	Overweight	Class 1	Class 2 or 3	Baseline prevalence
Normal weight					35%
Age < 25	85%	14%	∗	∗	22%
Age 25–49	82%	16%	1%	∗	40%
Age 50–74	84%	14%	2%	∗	29%
Age > 75	84%	14%	∗	∗	8%
Overweight					33%
Age < 25	23%	61%	16%	∗	9%
Age 25–49	14%	70%	14%	2%	43%
Age 50–74	11%	77%	11%	1%	39%
Age > 75	16%	71%	14%	∗	8%
Class 1					20%
Age < 25	∗	27%	53%	14%	8%
Age 25–49	∗	18%	67%	14%	44%
Age 50–74	∗	17%	70%	12%	41%
Age > 75	∗	24%	67%	∗	7%
Class 2 or 3					12%
Age < 25	∗	∗	18%	76%	9%
Age 25–49	∗	3%	17%	80%	49%
Age 50–74	∗	∗	17%	80%	38%
Age > 75	∗	∗	∗	64%	4%
Follow-up prevalence	35%	33%	20%	13%	

^∗^Cell count 20 or less.

## Data Availability

The datasets analyzed during the current study are publicly available in the MEPS repository “https://www.ahrq.gov/research/data/meps/index.html”.

## Abbreviations

BMI: Body mass index
CDC: Centers for Disease Control and Prevention
DPP: Diabetes Prevention Program
IDF: International Diabetes Federation
MEPS: Medical Expenditure Panel Survey
NIH: National Institutes of Health
WHO: World Health Organization.

## Additional Points

*Implication*. Intervention strategies should be tailored by age group. Younger patients are typically more weight mobile and may require less intensive intervention than older patients.
